# Impact of L-carnitine supplementation on gastric emptying and bowel function in pediatric ketogenic diet therapy: a clinical trial

**DOI:** 10.1038/s41598-024-78779-4

**Published:** 2024-11-15

**Authors:** May Fouad Nassar, Mennatallah Osama Shata, Shrouk Mohamed Awadallah, Mennatallah Ayman Youssef, Haya Essam Ibrahim

**Affiliations:** 1 Pediatric Clinical Nutrition Unit, Department of Pediatrics, Faculty of Medicine, Ain Shams University, Cairo, Egypt; 2 Pediatric Neurology Unit, Department of Pediatrics, Faculty of Medicine, Ain Shams University, Cairo, Egypt; 3 Department of Radiology, Faculty of Medicine, Ain Shams University, Cairo, Egypt

**Keywords:** Clinical trial, Ketogenic diet, Pediatric drug resistant epilepsy, L-carnitine, Gastric emptying, Neuroscience, Medical research, Neurology

## Abstract

Ketogenic diet (KD) is an excess fat, enough protein, and minimal carbohydrate diet. The high fat content in KD lowers the oesophageal sphincter tone, slows gastric emptying, and decreases intestinal transit time. The primary aim of the current clinical trial was to study the effect of L-carnitine supplementation on gastric emptying in children with drug resistant epilepsy (DRE) on KD. Assessment of the protective effect of L-carnitine on bowel function and habits in those patients was a secondary aim. The current study recruited 30 patients aged 12 months to 18 years newly diagnosed with DRE assigned to start KD who were following up at the Pediatric Clinical Nutrition and Neurology Outpatient Clinics or were admitted due to DRE at the Pediatric Neurology Inpatient Department, Children’s Hospital, Ain Shams University (Egypt). Participants were assigned randomly into 2 arms; arm I: received KD with L-carnitine supplementation, arm II: received KD only. Patients were assessed at baseline and after 3 months of starting KD, the assessments of children included: 24-hour dietary recall, Chalfont Seizures Severity Scale, gastrointestinal symptoms score and Bristol stool chart, frequency of defecation per week, anthropometric measurements assessment, fasting serum lipid profile and measurement of the antral length by ultrasound. There was significant increase in antral length in the patients who received KD with L-carnitine supplementation compared to the non-supplemented group. The antral length showed a significant negative correlation with GI symptoms score in all cases and the L-carnitine supplemented group. It also showed a significant positive correlation with Bristol stool score in all patients and a significant positive correlation with stool frequency in the L-carnitine supplemented group only. L-carnitine supplementation to children with DRE on KD has a significant role in improving gastric motility and it increases the frequency of defecation. Further studies are recommended to explore additional benefits, meanwhile it is prudent to advise L-carnitine supplementation for such patients.

## Introduction

Ketogenic diet (KD) is an excess fat, enough protein, and minimal carbohydrate diet which is administered under medical supervision to maintain chronic ketosis while providing adequate protein and calories for growth and development^1^. KD is an efficient treatment for pediatric drug-resistant epilepsy (DRE). The latter is defined as failure of adequate trials of two tolerated, appropriately chosen and used antiepileptic drug (AED) schedules (whether as monotherapies or in combination) to achieve sustained seizure freedom^2^. KD is more effective than several novel anticonvulsant medications and families of affected children accept it because of its tolerability as well as its efficiency^3^.

The high fat content in KD lowers the oesophageal sphincter tone, slows gastric emptying, and decreases intestinal transit time^4,5^.

Gastrointestinal disturbances, constipation, dyslipidemia, and micronutrient deficiencies are common side effects in the KD regimen which is often discontinued earlier than recommended because such symptoms are difficult to be controlled^6^.

Carnitine deficiency has been widely reported in patients with epilepsy being treated with valproic acid and there have been reports that carbamazepine and phenobarbital may also deplete carnitine levels especially if used in combination with valproic acid. It has been suggested that the KD may also induce carnitine deficiency due to the high fat content^7^.

In patients with severe intellectual and motor disabilities, Murata and collaborators reported that the level of free carnitine was significantly correlated with gastric emptying and the severity of constipation and this level was significantly lower in the patients who suffered from constipation. Moreover, the severity of constipation was significantly relieved after supplementation with carnitine^8^.

Gastric emptying is a complex physiologic process controlled by the physical and chemical composition, sympathetic and parasympathetic innervations of the stomach, and circulating neuro-endocrine transmitters. The type of food, volume, and caloric content significantly affect the rate of gastric emptying^9^.

Gastric emptying can be assessed by ultrasonography which is a non-invasive technique that can be repeatedly performed because of its safety with good correlation to radionuclide estimates of gastric emptying^10^.

## Aim of the work

Primary aim: Assessment of the effect of L-carnitine supplementation on gastric emptying in pediatric patients with DRE on KD through measuring antral length.

Secondary aim: Assessment of the protective effect of L-carnitine on bowel function and habits in patients with DRE on KD by assessing frequency of defecation, stool form and response to laxatives.

## Patients and methods

### Setting

This randomized controlled clinical trial study was conducted on 30 patients who presented to the Pediatric Clinical Nutrition and Neurology Units, Children`s Hospital, Faculty of Medicine, Ain Shams University (Cairo, Egypt), during the period from January 2023 to November 2023. These units run a multidisciplinary clinic for children with DRE which means failure of two or more well selected and proper dosed AED to achieve sustained seizure freedom^2^. This clinic supports the use of KD for children with DRE.

### Ethical considerations

Written informed consent was obtained from all children’s guardians. The study was performed according to The Code of Ethics of the World Medical Association (Declaration of Helsinki) for studies involving humans.

This study was conducted after approval by the Research Ethical Committee of Faculty of Medicine, Ain Shams University with approval number (FMASU M S 630/2022) in 22/6/2022. The study was also registered as a clinical trial [ClinicalTrials.gov Identifier NCT06255873 (13/02/2024)]. The CONSORTreporting guidelines was used to present the clinical trial data^11^.

### Patients

The current study recruited 30 patients aged 12 months to 18 years newly diagnosed with DRE assigned to start KD who were following up at Pediatric Clinical Nutrition and Neurology Outpatient Clinics or were admitted due to drug resistant seizures at Pediatric Neurology inpatient department.

Patients with intolerance or non-compliance to ketogenic diet therapy (KDT), patients who fit the criteria for epilepsy surgery or vagal nerve stimulation, patients with history of previous abdominal operations or gastrointestinal (GI) diseases affecting GI motility before starting KD, and patients with tube feeding or feeding gastrostomy or jejunostomy were excluded from the study.

Participants were assigned randomly by the field researchers using simple randomization with 15 patients in each group using a shuffled deck of cards (even–group arm I, odd–group arm II) into 2 arms; arm I patients received KD with L-carnitine supplementation and arm II patients received only KD.

All Patients started KDT under supervision of physicians. Ketogenic ratio was assigned {total fat intake (gram) divided by the sum of total protein intake (gram) and total carbohydrate intake (gram)}.

The type and initial ratio of KD therapy were selected according to age, type of feeding, needs and the preferences of the child and his/her family were considered. KD ratio ranged from 1.5:1 to 3:1 of lipids to carbohydrates plus proteins, in grams^12^.

L-carnitine supplementation in arm I patients was taken after feed in the form of tablets or dissolved tablets with a daily dose of 50 mg/kg/day, up to a maximum of 2 g/day, once per day^8,13^.

Side effects as nausea, diarrhea, and fishy body odor^13^ were monitored.

Every case from arm I was matched for gender, age (1 year range was allowed between the selected patients), and BMI Z score (1 SD range was allowed between them) with a patient from arm II to serve as controls.

Sample size: Thirty patients were enrolled. Using jepi-info program for sample size calculation and reviewing results from previous study^8^, sample size of 30 patients were sufficient to explore and achieve the study objectives. Patients assigned randomly into 2 groups 15 patients in each group.

## Methods

All the data mentioned below was obtained from both groups at the initiation of KD and after 3 months of intervention and all enrolled patients completed the follow up assessment.

Detailed medical history was taken with special emphasis on age of onset and duration of the disease, frequency of epileptic attacks and number of hospital admissions due to severe attacks, as well as history of antiepileptic drugs (AEDs) (drug name, number of drugs, dosage, and duration of therapy). Dietary assessment using 24-hour recall was obtained and analyzed using software based on food composition tables of the National Nutrition Institute, 2006^14^.

Complete physical and neurological examination were performed laying stress on anthropometric measurements [weight (kg), height or length (cm) and body mass index (kg/m^2^)] which were measured and plotted on the WHO Z score for children up to 5 years of age^15^, while CDC 2000 growth charts have been used for those above 5 years of age.

Gastro-intestinal symptoms score was assessed based on the score of Adam and collaborators^16^ as follows:

Parents were asked to indicate whether their children had experienced specific GI symptoms nausea, vomiting, bloating, abdominal cramps, early satiety, eructation or heartburn, loss of appetite, retrosternal discomfort and epigastric pain or abdominal pain over the past month. Children or their care givers were asked to rate the intensity of each individual symptom on a validated 5-point Likert scale (0 indicates no problem, 1 indicates mild problem, 2 indicates moderate problem, 3 indicates severe problem and 4 indicates very severe problem). The sum score ranged from (0–40). Score of (1–10) indicates mild problem, (11–20) indicates moderate problem, (21–30) indicates severe problem, (31–40) indicates very severe problem^16^.

Constipation was assessed by frequency of defecation, response to laxatives and feces forms which were classified according to Bristol stool scale, (type 1 = separate hard lumps) (type 2 = sausage shaped and lumpy) (type3 = sausage shaped with cracks on its surface) (type 4 = sausage or snake with smooth surface) (type 5 = soft blobs) (type 6 = fluffy pieces) (type 7 = entirely liquid)^17^.

 Severity of seizures was assessed using the Chalfont seizure severity scale (CSSS)^18^.

### Radiological assessment

Initial pelvi-abdominal ultrasound (PAUS) using a GE healthcare LOGIQ P9 ultrasound device was done 15 min after a meal of KD milk formula containing 25% of the recommended dietary allowance (RDA) of daily calories for each patient^19^. The same operator performed all the ultrasound studies.

All patients were studied in a supine position, measurements of the gastric antrum were always taken from the outer profile of the wall because the lumen of the antrum is usually very narrow, the cross section of the gastric antrum was measured, corresponding to the antral diameter, (forms an elliptical shape). The longitudinal area was measured representing antral length, as described by Bolondi and collaborators in 1985^20^.

### Laboratory assessment

Specimen Collection: Blood collection was performed with care to avoid stasis, hemolysis or contamination by tissue fluids, or exposure to glass. Specimens were kept at room temperature. Laboratory tests: Fasting Lipid profile was assessed after 10 h of fasting, and dyslipidemia was defined as the presence of any of the following four measurements: high total-cholesterol (≥ 200 mg/dL), high LDL-cholesterol (≥ 130 mg/dL), low HDL-cholesterol (≤ 35 mg/dL), or high triglycerides (≥ 130 mg/dL)^21^.

### Statistical analysis

The collected data was coded, processed, and analyzed using the SPSS (Statistical Package for Social Sciences) version 27 for Windows^®^ (IBM SPSS Inc, Chicago, IL, USA). Categorical variables were conveyed as frequency with percentage while continuous variables were described as mean and standard deviation (SD) or median with interquartile range (IQR). Quantitative data were tested for normality by Kolmogorov-Smirnov test. The categorical variables were contrasted utilizing Chi-square test or Fisher’s exact test. To compare continuous variables among groups, Student’s t test, Mann-Whitney U test and Friedman test were utilized. The statistical significance of the difference between two means assessed twice for the same research group was evaluated using a paired t-test or and Wilcoxon signed-rank test. P value *≤* 0.05 was considered significant.

## Results

The current study comprised 30 patients with DRE who completed the 3 months clinical trial. There were no major side effects that led to withdrawal from the clinical trial prior to the last assessment. Enrolled patients were divided into two groups. Each group included 15 patients and only patients in group one (arm I) received L-carnitine supplementation in addition to KDT.

Regarding CSSS, there was a significant decrease in CSSS in all studied patients after intervention (the range decreased from an initial of 19–118 to 10–99 after intervention with a P-value of 0.025). Nevertheless, comparison between the two studied groups after 3 months follow up revealed no significant difference (P-value = 0.097).

As regards the anthropometric measurements, the current study showed significant increase in weight z-score and height z-score with non-significant change in BMI z-score after 3 months of KD initiation (with P-values of 0.002, 0.002 and 0.06 respectively).

Significant increase in antral length in the patients who received KD with L-carnitine supplementation after intervention is demonstrated in Table 1. On the other hand, there was no significant difference regarding antral length in the patients who only received KD upon follow up (P-value 0.925).

**Table 1:** Comparison between the patients received KD with L-carnitine supplementation at time of enrollment and after intervention regarding the antral length assessed by ultrasound.

		L- carnitine supplementation group		Test-value	P-value	Sig
		Before intervention No. = 15	After intervention No. = 15			
Antral length (cm)	Mean ± SD	1.85 ± 0.40	2.16 ± 0.69	**-2.273**	**0.039**	**S**
	Range	1.24–2.57	1.09–3.38			

*S* Significant.

Additionally, Table 2 shows a significant negative correlation between the antral length by ultrasound and GI symptoms score in all cases and the group who received KD with L-carnitine supplementation indicating that an increase in antral length was associated with clinical improvement of GI symptoms. Additionally, there was a significant positive correlation between the antral length by ultrasound and Bristol stool score in all cases and a distinct positive (almost significant) with the group who received KD with L-carnitine supplementation indicating that an increase in antral length was associated with higher Bristol stool score hence a softer stool consistency. Also, there was a significant positive correlation between the antral length by ultrasound and frequency of defecation in KD with L-carnitine supplementation group only after intervention.

**Table 2:** Correlation between antral length by ultrasound and GI symptoms score, Bristol stool scale and frequency of defecation after intervention.

	FU antral length (cm)					
	All studied patients		L-carnitine supplementation group		KD only group	
	*r*	*P*-value	*r*	*P*-value	*r*	*P*-value
FU GI symptoms` score	− 0.471	**0.009**	− 0.592	**0.020**	− 0.279	0.313
FU Bristol stool score	0.435	**0.016**	0.488	0.065	0.410	0.129
FU frequency of defecation per week	0.354	0.055	0.629*	**0.012**	0.027	0.924

*FU* follow up, *GI* gastrointestinal.

Worth noting here is that no significant difference was detected between the two studied groups regarding initial laxative use. Additionally, the correlation studies showed no significant correlations between the KD ratio and frequency of defecation, Bristol stool scale, vomiting score, GI symptoms` score and antral length after intervention in all patients.

Regarding 24-hour dietary recall Table 3 revealed that there was a positive correlation between fibers content in 24 h dietary recall and antral length after intervention in KD with L-carnitine supplementation group indicating that an increase in fibers content in 24 h dietary recall was associated with higher antral length, but there was no correlation between antral length by ultrasound and KD ratio or the other micronutrients. On the other hand, there was a significant negative correlation between GI symptoms score and 24 h dietary contents of potassium and fibers with P-values of 0.027 and 0.016 (respectively), that indicates the higher potassium or fibers dietary contents, the lower the GI symptoms score.

**Table 3:** Correlation between antral length by ultrasound and 24 h dietetic recall including KD ratio and micronutrients after intervention.

	FU antral length (cm)					
	All cases		L-Carnitine supplementation group		KD only group	
	*r*	*P*-value	*r*	*P*-value	*r*	*P*-value
KD ratio	− 0.329	0.076	− 0.451	0.091	− 0.151	0.592
Sodium (Na) (mg/day)	0.190	0.314	0.368	0.177	0.032	0.909
Phosphorus (PO_4_) (mg/day)	0.040	0.834	0.202	0.470	0.016	0.955
Potassium (K) (mg/day)	0.341	0.065	0.466	0.080	0.242	0.386
Calcium (Ca) (mg/day)	− 0.033	0.863	0.073	0.795	− 0.184	0.511
Water (ml/day)	0.288	0.122	0.458	0.086	0.193	0.490
Zinc (Zn) (mg/day)	0.228	0.226	0.229	0.412	0.236	0.397
Iron (mg/day)	0.321	0.084	0.447	0.095	0.211	0.450
Fibers (gm/day)	0.342	0.064	0.633*	**0.011**	0.030	0.914

*FU* follow up, *KD* ketogenic diet.

As shown in Table 4, there was highly significant increase in the of frequency of defecation in KD with L-carnitine supplementation group by 50% in comparison to KD only group that showed decrease in frequency of defecation per week by 33%. Although there was a decrease in the median (IQR) of GI symptoms score in the patients on KD with L-carnitine supplementation in comparison to KD only group indicating clinical improvement in GI symptoms, and an increase in the median (IQR) of the Bristol stool scale indicating that the stool was softer, these results didn’t reach statistical significance.

**Table 4:** Comparison between the two studied groups regarding the rate of change in GI symptoms score, Bristol stool scale and frequency of defecation after intervention.

Rate of change %		L-Carnitine supplementation group	KD only group	Test value	P-value	Sig
		No. = 15	No. = 15			
GI symptoms score	Median (IQR)	− 28.6 (− 50.0 to 0.0)	21.4 (− 33.3 to 45.5)	− 1.663	0.098	NS
	Range	− 57.14 to 62.5	− 57.14 to 66.67			
Bristol stool scale	Median (IQR)	25.0 (− 25.0 to 66.7)	0.0 (− 25.0 to 33.3)	− 1.065	0.305	NS
	Range	− 40 to 200	− 50 to 100			
Frequency of defecation (/week)	Median (IQR)	50.0 (0.0 to 100.0)	− 33.3 (− 40.0 to 0.0)	− 3.186	**0.001**	HS
	Range	− 50 to 200	− 55.56 to 66.67			

*GI* gastrointestinal, *NS* Non-significant, *HS* Highly significant, *LDL* low density lipoprotein, *HDL*, high density lipoprotein, *TG*, triglycerides, *NS* Non-significant, *HS* Highly significant.

Considering serum lipid profile result, Table 5 shows there was highly significant increase in HDL levels after intervention in the L-carnitine supplemented group, while serum cholesterol, LDL and triglycerides results were comparable before and after intervention.

**Table 5:** Comparison between the patients received KD with L-carnitine supplementation regarding fasting lipid profile at time of enrolment and after intervention.

		L-Carnitine supplementation group		Test-value	P-value	Sig
		Before	After			
		No. = 15	No. = 15			
Cholesterol in mg/dl	Mean ± SD	178.60 ± 67.20	193.53 ± 74.64	− 1.796	0.094	NS
	Range	109–360	113–380			
LDL in mg/dl	Mean ± SD	98.20 ± 35.76	109.07 ± 44.34	− 1.544	0.145	NS
	Range	53–165	60–229			
HDL in mg/dl	Mean ± SD	57.40 ± 19.57	63.40 ± 19.07	3.994	**0.001**	HS
	Range	32–40	38–60			
TG in mg/dl	Mean ± SD	94.03 ± 36.61	103.53 ± 24.76	− 1.806	0.093	NS
	Range	50–189	65–148			

## Discussion

The aim of the current study was to assess the effect of implementing L-carnitine as an adjuvant therapy with KD on gastric emptying in children with DRE, and to assess its protective effect of L-carnitine on bowel function and habits. Previous data is scarce in this domain; thus, the current study can be considered a pioneer in studying the effect of L-carnitine on gastro-intestinal symptoms in pediatric patients on KDT.

The significant improvement in CSSS in our series of studied DRE patients on KDT comes in agreement with several previous research. Recently, El-Rashidy and co-researchers reported a significant decrease in the severity of the seizure after KDT^22^. Similar finding was reported by Nassar and collaborators who studied twenty-one patients on KDT, and their results showed more than 50% reduction in their seizures^23^.

The fact that L-carnitine supplementation didn`t add to the patients` seizure control was previously reported by Selter and collaborators who studied carnitine supplementation as one of dietary modifications to improve seizure control in patients on KDT and reported that this dietary modification wasn`t ideal for improving seizure control^24^. On the other hand, the research of Fukuda and co-researchers showed that there was a significant negative correlation between blood ammonia and free carnitine concentrations in epileptic children (*P* = 0.003) and this high serum level of ammonia was associated with increased severity of seizures in their series of patients^25^. The work of the latter authors has opened the field for further research regarding the role of L-carnitine supplementation in seizure control.

Regarding L-carnitine supplementation, the current results showed that there was a decrease in the median (IQR) of GI symptoms score in the patients on KD with L-carnitine supplementation in comparison to KD only group indicating clinical improvement in GI symptoms. There was also an increase in the median (IQR) of the Bristol stool scale indicating that the stools were softer. These results although distinct, didn’t reach statistical significance. Nevertheless, the current study showed a highly significant increase in frequency of defecation (50%) in KDT with L-carnitine supplementation group in comparison to 33% in KD only group.

In 2017, Murata and collaborators reported that serum carnitine level was negatively correlated with severity of constipation in 27 patients; age range, 2 to 45 years with severe motor and intellectual disabilities, and it was significantly lower in the constipation group compared to the non-constipation group^8^. This was attributed to the involvement of L-carnitine in mitochondrial transport of fatty acids thus its deficiency affects multiple organs including the smooth muscles in the gastrointestinal tract. Murata and coresearchers also added that infants on a diet deficient in carnitine, had gastrointestinal dysmotility manifested by postprandial vomiting, oral drooling and delayed gastric emptying and infrequent bowel movements^8^.

As regards antral length, the current results revealed a significant increase in antral length in the patients who received KD with L-carnitine supplementation after intervention in comparison to the time of enrolment, while there was no significant change noticed in the non-supplemented group. Additionally, there was a negative correlation between antral length and GI symptoms score in all studied patients and the group who received KD with L-carnitine supplementation indicating that an increase in antral length was associated with clinical improvement of GI symptoms. This could be explained by the fact that longitudinal smooth muscles contractions cause increase in its length^26^ these contractions improve gastric emptying and provide proper food mixing.

A significant positive correlation between enteral length and Bristol stool score was demonstrated in all studied patients and a distinct positive one in the group who received KD with L-carnitine supplementation indicating that an increase in antral length was associated with higher Bristol stool score thus a softer stool consistency. Moreover, a positive correlation between the antral length and frequency of defecation per week was found in KD with L- carnitine supplementation group denoting that an increase in antral length was associated with more frequent defecations.

A significant positive correlation between fiber content in 24 h dietetic recall and antral length after intervention in KD with L-carnitine supplementation group indicating that an increase in fiber content in 24 h dietary recall was associated with higher antral length. Contrary to these results, Gill and collaborators, showed that dietary fibers increase gut luminal viscosity and consumption of viscous dietary fibers, alter transit time in the upper gut, including decreasing gastric emptying rate and modulating small intestinal transit^27^. Nevertheless, the current results can be supported by the previous literature data on fifty-six chronically constipated children (not on KDT) which showed that the children who received cocoa husk (fibers) supplements tended to increase the number of bowel movements more than the children of the placebo group^28^.

Inadequate fiber intake in children on KDT is poorly understood and could go beyond constipation and gastrointestinal discomfort^29^. Moreover, a study by Lopez and collaborators found that patients with constipation tended to have higher incidence of hypokalemia but these results didn’t reach statistical significance^30^. In this domain, the current study revealed a significant negative correlation between GI symptoms scores and 24 h dietary contents of potassium and fibers in all studied patients. These results signify that the higher potassium and fibers dietary contents, the lower the GI symptoms score which indicates clinical improvement. Although, only scarce data had been found to highlight the correlation between 24 h macronutrient or micronutrients dietary contents of patients on KDT and GI symptoms, the current results highlight the importance of dietary potassium and fibers for better gut function.

On studying the effect of L-carnitine on fasting lipid profile, our study showed that the patients who received KD with L-carnitine supplementation showed highly significant increase in HDL level after intervention. On the other hand, the comparison between both groups regarding Cholesterol, LDL and TG levels showed no significant difference at time of enrolment and after intervention. This protective effect of L-carnitine is of utmost importance since increase in total cholesterol and triglyceride concentrations upon KD therapy has been previously reported in children^21^. Moreover, the present study agrees with a study performed on 25 children with type 1 diabetes mellitus and revealed that L- carnitine supplementation caused a significant decrease in serum total cholesterol and LDL cholesterol and showed a significant increase in HDL serum levels^31^.

Finally, regarding the results of anthropometric measurements, the present study showed significant increase in weight z-score and height z-score with non-significant change in BMI z-score after 3 months of KD initiation. These results agree with El-Rashidy and collaborators who reported that there was significant increase in the weight, z-score for weight, height, and z score for height with non-significant change in BMI and BMI z-score after KD treatment^22^. Additionally, Nassar and co-researchers reported that there was non-significant increase in z-score for weight, z-score for height and BMI after 6 months of KD. Thus, we highlight the importance of maintaining proper physical growth assessment during KDT^23^.

In conclusion, although L-carnitine supplementation for pediatric DRE didn`t improve the seizure control during KDT, this supplementation improved stool consistency and increased the frequency of defecation. There was a positive correlation between antral length and improvement of the gastro-intestinal symptoms in those patients. Additionally, L-carnitine supplementation has a positive effect on fasting lipid profile since it led to a significant increase in HDL levels in pediatric patients on KDT. Further longer term, larger scale and preferably multi-center studies are recommended to explore additional benefits, meanwhile it is prudent to advise L-carnitine supplementation for such patients.

### Study limitations

Although the current findings are consistent and coherent, strongly indicating the external validity of the study, we do recommend future preferably large prospective clinical trials, to increase their internal validity.

The current study has its own limitations, no comparisons were made with patients on traditional AEDs or placebo and the short follow up duration are among these limitations.

## Data Availability

‘The datasets generated and /or analyzed during current study available from the corresponding author upon reasonable request.

## References

[1] Sharma, S., Sankhyan, N., Gulati, S. & Agarwala, A. Use of the modified Atkins diet for treatment of refractory childhood epilepsy: a randomized controlled trial. *Epilepsia*. **54** (3), 481–486 (2013). 23294191 10.1111/epi.12069

[2] Mishra, P., Singh, S. C. & Ramadass, B. Drug resistant epilepsy and ketogenic diet: a narrative review of mechanisms of action. *World Neurosurg. X*. **25**, 22:100328. 10.1016/j.wnsx.2024.100328 (2024). 10.1016/j.wnsx.2024.100328 PMC10914588 38444870

[3] Freeman, J. M. et al. The efficacy of the ketogenic diet-1998: a prospective evaluation of intervention in 150 children. *Pediatrics.* **102**(6), 1358–1363. 10.1542/peds.102.6.1358 (1998). 10.1542/peds.102.6.1358 9832569

[4] McArtney, R., Bailey, A. & Champion, H. What is a ketogenic diet and how does it affect the use of medicines? *Arch. Dis. Child. Educ. Pract. Ed.* **102**, 194–199. 10.1136/archdischild-2014-307000 (2017). 27469127 10.1136/archdischild-2014-307000

[5] Weisbrodt, N. W., Wiley, J. N., Overholt, B. F. & Bass, P. A relation between gastroduodenal muscle contractions and gastric emptying. *Gut*. **10** (7), 543–548. 10.1136/gut.10.7.543 (1969). 5806934 10.1136/gut.10.7.543 PMC1552942

[6] Poelzer, K., Mannion, C., Ortiz, M. M., Bang, R. & Woods, P. A systematic review of the quality of life for families supporting a child consuming the ketogenic diet for seizure reduction. *Curr. Dev. Nutr.* **3** (5), 104–108. 10.1093/cdn/nzy079 (2019). 10.1093/cdn/nzy079 PMC6488515 31044188

[7] Nael, E. G., Zupec-kania, B. & Pfeifer, H. H. Carnitine, nutritional supplementation, and discontinuation of ketogenic diet therapies. *Epilepsy Res.* **100** (3), 267–271. 10.1016/j.eplepsyres.2012.04.021 (2012). 22704584 10.1016/j.eplepsyres.2012.04.021

[8] Murata, S., Inoue, K., Amoatsu, T., Yoden, A. & Tamai, H. Supplementation with carnitine reduces the severity of constipation: a retrospective study of patients with severe motor and intellectual disabilities. *J. Clin. Biochem. Nutr.* **60** (2), 121–124. 10.3164/jcbn.16-52 (2017). 28366991 10.3164/jcbn.16-52 PMC5370531

[9] Farrell, M. B. Gastric emptying scintigraphy. *J. Nucl. Med. Technol.* **47** (2), 111–119. 10.2967/jnmt.117.227892 (2019). 31167827 10.2967/jnmt.117.227892

[10] Steinsvik, E. K., Hatlebakk, J. G., Hausken, T., Nylund, K. & Gilja, O. H. Ultrasound imaging for assessing functions of the GI tract. *Physiol. Meas.* **42** (2), 024002. 10.1088/1361-6579/abdad7 (2021). 33434898 10.1088/1361-6579/abdad7

[11] Schulz, K. F., Altman, D. G., Moher, D. & for the CONSORT Group. CONSORT 2010 Statement: updated guidelines for reporting parallel group randomised trials. 10.3736/jcim20100702 20619135

[12] Luz, I. et al. Ketogenic diet for refractory childhood epilepsy: beyond seizures control. The experience of a Portuguese Pediatric Centre. *Portuguese Pediatr. Centre*. **32** (12), 760–766. 10.20344/amp.12184 (2019). 10.20344/amp.12184 31851885

[13] De Vivo, D. C. et al. L-carnitine supplementation in childhood epilepsy: current perspectives. *Epilepsia*. **39** (11), 1216–1225. 10.1111/j.1528-1157.1998.tb01315.x (1998). 9821988 10.1111/j.1528-1157.1998.tb01315.x

[14] National institute of Nutrition. *Food Composition Tables for Egypt*. 2nd edn. (2006).

[15] World Health Organization. *WHO Child Growth Standards: Length/height-for-age, Weight-for-age, Weight-for-length, Weight-for-height and Body Mass Index-for-Age: Methods and Development* (World Health Organization, 2006).

[16] Adam, B., Liebregts, T., Saadat-Gilani, K., Vinson, B. & Holtmann, G. Validation of the gastrointestinal symptom score for the assessment of symptoms in patients with functional dyspepsia. *Aliment Pharmacol. Ther.* **22**(4), 357–63. 10.1111/j.1365-2036 (2005). 10.1111/j.1365-2036.2005.02572.x 16098003

[17] Lewis, S. J. & Heaton, K. W. Stool form scale as a useful guide to intestinal transit time. *Scand. J. Gastroenterol.* **32** (9), 920–924. 10.3109/00365529709011203 (1997). 9299672 10.3109/00365529709011203

[18] Duncan, J. S. & Sander, J. W. The Chalfont seizure severity scale. *JNNP*. **54** (10), 873–876. 10.1136/jnnp.54.10.873 (1991). 10.1136/jnnp.54.10.873 PMC1014570 1744641

[19] Havel, R. et al. *Recommended Dietary Allowances*, 10th edn, vol. 10, 24–38 (The National Academies Press, 1989). 25144070

[20] Bolondi, L. et al. Measurement of gastric emptying time by real-time ultrasonography. *Gastroenterology*. **89** (4), 752–759. 10.1016/0016-5085(85)90569-4 (1985). 3896910 10.1016/0016-5085(85)90569-4

[21] Yılmaz, Ü. et al. The effect of ketogenic diet on serum lipid concentrations in children with medication resistant epilepsy. *Seizure*. **91**, 99–107. 10.1016/j.seizure.2021.06.008 (2021). 34147890 10.1016/j.seizure.2021.06.008

[22] El-Rashidy, O. F., Nassar, M. F., Shokair, W. A. & El Gendy, Y. G. A. Ketogenic diet for epilepsy control and enhancement in adaptive behavior. *Sci. Rep.* **13** (1), 2102. 10.1038/s41598-023-27373-1 (2023). 36747012 10.1038/s41598-023-27373-1 PMC9902473

[23] Nassar, M. F., El-Gendy, Y. G. A., Hamza, M. T., Mohamed, M. N. & Radwan, N. Effect of ketogenic diet for drug–resistant epilepsy on immunological cells. *Neurol. Sci.* **43**, 1987–1992. 10.1007/s10072-021-05574-8 (2022). 34449000 10.1007/s10072-021-05574-8

[24] Selter, J. H., Turner, Z., Doerrer, S. C. & Kossoff, E. H. Dietary and medication adjustments to improve seizure control in patients treated with the ketogenic Diet. *J. Child. Neurol.* **30** (1), 53–57. 10.1177/0883073814535498 (2015). 24859788 10.1177/0883073814535498 PMC4241191

[25] Fukuda, M. et al. Carnitine deficiency: risk factors and incidence in children with epilepsy. *Brain Dev.* **37** (8), 790–796. 10.1016/j.braindev.2014.12.004 (2015). 25547040 10.1016/j.braindev.2014.12.004

[26] Szurszewski, J. H. Mechanism of action of pentagastrin and acetylcholine on the longitudinal muscle of the canine antrum. *J. Physiol.* **252** (2), 335–361. 10.1113/jphysiol.1975.sp011147 (1975). 1520 10.1113/jphysiol.1975.sp011147 PMC1348448

[27] Gill, S. K., Rossi, M., Bajka, B. & Whelan, K. Dietary fiber in gastrointestinal health and disease. *Nat. Rev. Gastroenterol. Hepatol.* **18** (2), 101–116. 10.1038/s41575-020-00375-4 (2021). 33208922 10.1038/s41575-020-00375-4

[28] Castillejo, G., Bullo, M., Anguera, A., Escribano, J. & Salas-Salvado, J. A controlled randomized double-blind trial to evaluate the effect of a supplement of cocoa husk that is rich in dietary fiber on colonic transit in constipated pediatric patients. *Pediatrics*. **118** (3), e641–e648. 10.1542/peds.2006-0090 (2006). 16950955 10.1542/peds.2006-0090

[29] Dahl, W. J., Niebergall, E. J., Owen, R. J. Infant implications of fiber inadequacy in the ketogenic diet: A case study. *ICAN Child Adolesc. Nutr.* **3**(5), 288–290. 10.1177/1941406411422253 (2011).

[30] Lopez, J. et al. Constipation in the critically ill child: frequency and related factors. *J. Pediatr.* **167** (4), 857–861. 10.1016/j.jpeds.2015.06.046 (2015). 26254837 10.1016/j.jpeds.2015.06.046

[31] Badreldeen, A., El Razaky, O., Erfan, A., El-Bendary, A. & El Amrousy, D. Comparative study of the efficacy of captopril, simvastatin, and L-carnitine as cardioprotective drugs in children with type 1 diabetes mellitus: a randomised controlled trial. *Cardiol. Young*. **31** (8), 1315–1322. 10.1017/S1047951121000226 (2021). 33536102 10.1017/S1047951121000226

